# Supplementary material for: Comparison of Localization Methods in Cushing Disease—Could [11C]C-Methionine PET/CT Replace MRI or BIPSS?
Source: Cancers (Basel). 2025 Sep 27;17(19):3147. doi: 10.3390/cancers17193147 (PMC12523277; doi:10.3390/cancers17193147)
Supplement: Supplementary file 1 [file cancers-17-03147-s001.zip › cancers-3870088-supplementary.pdf]

Supplementary Figures

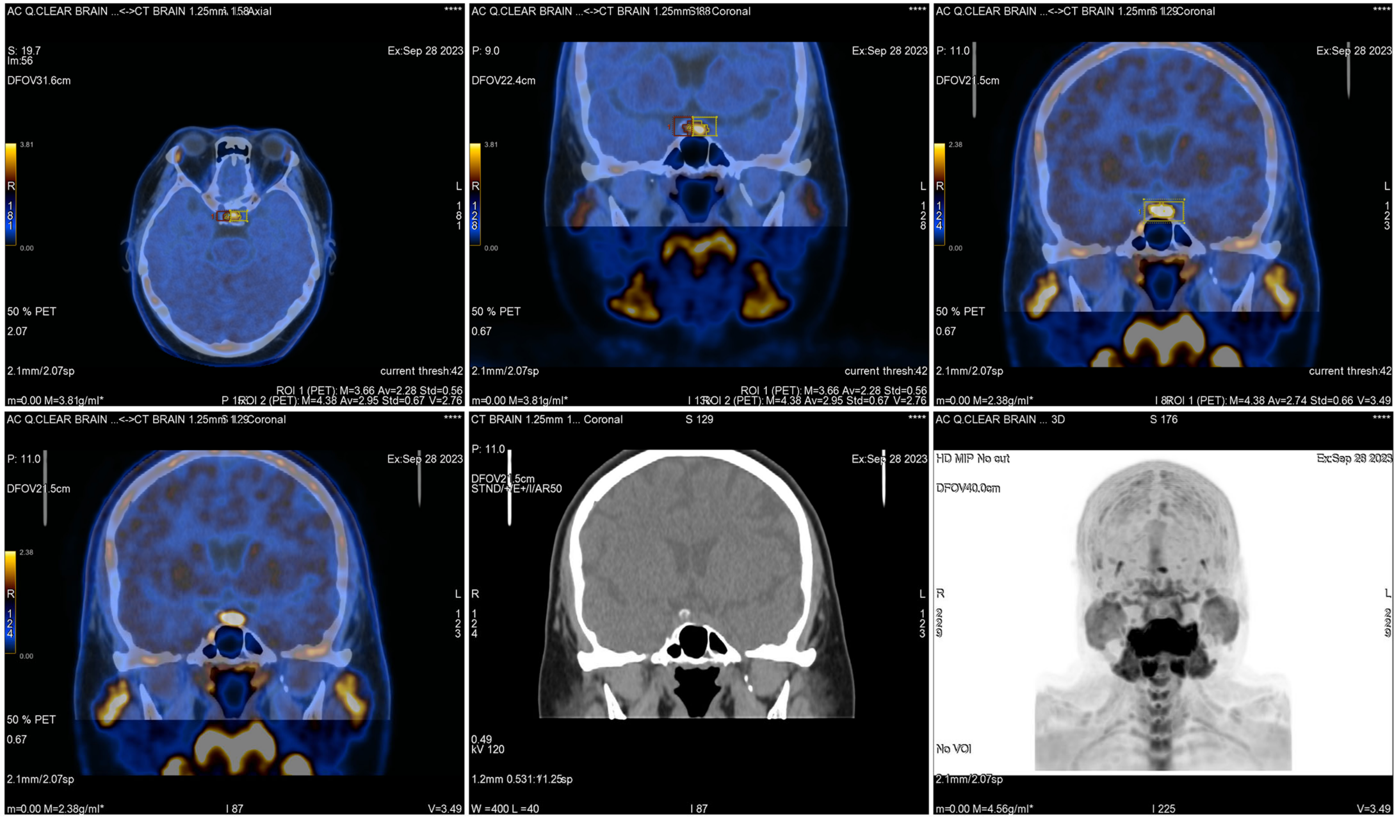

Suppl. Figure S1. [^{11}C]C-MET PET/CT study - True positive (Patient No. 1)

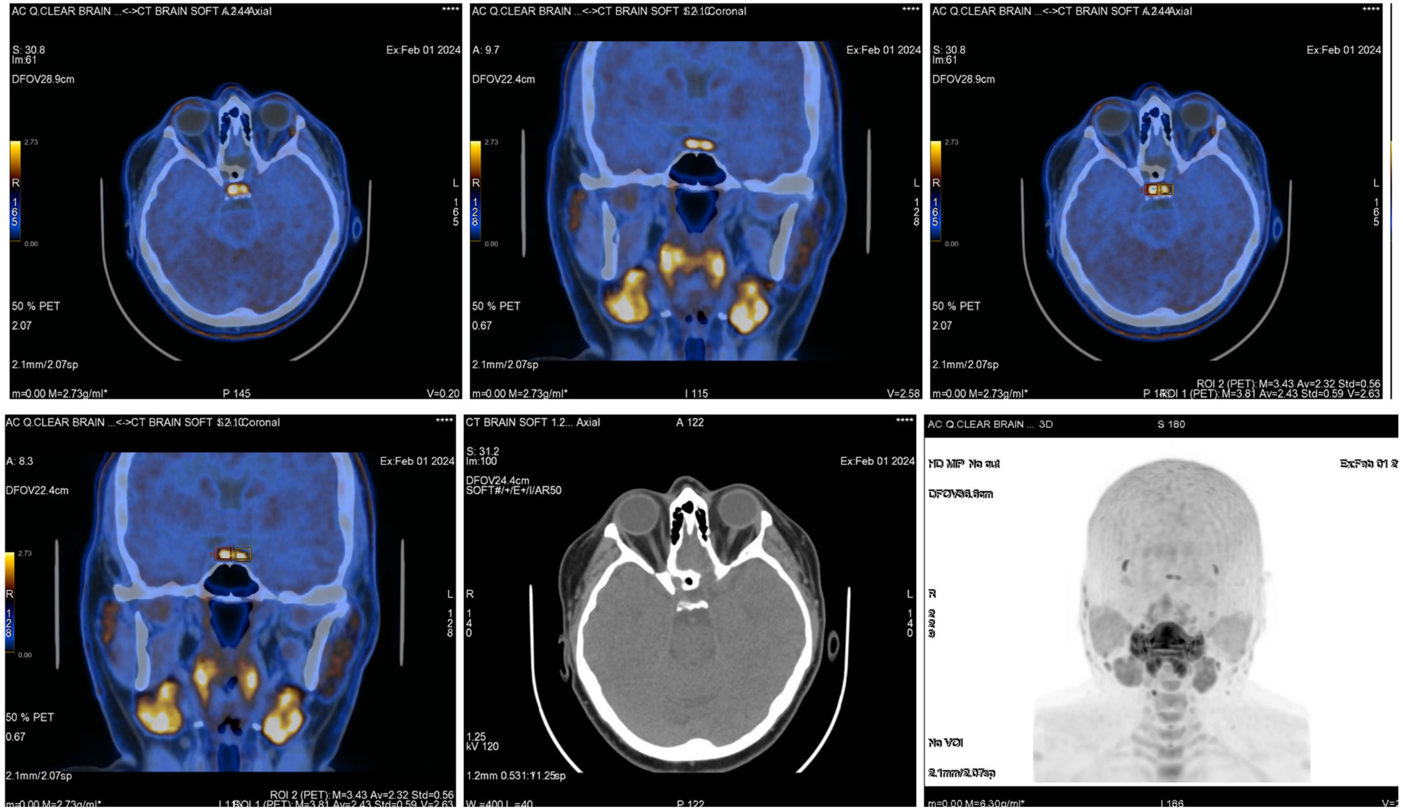

Suppl. Figure S2. [11C]C-MET PET/CT study - True negative. (Patient No. 8)

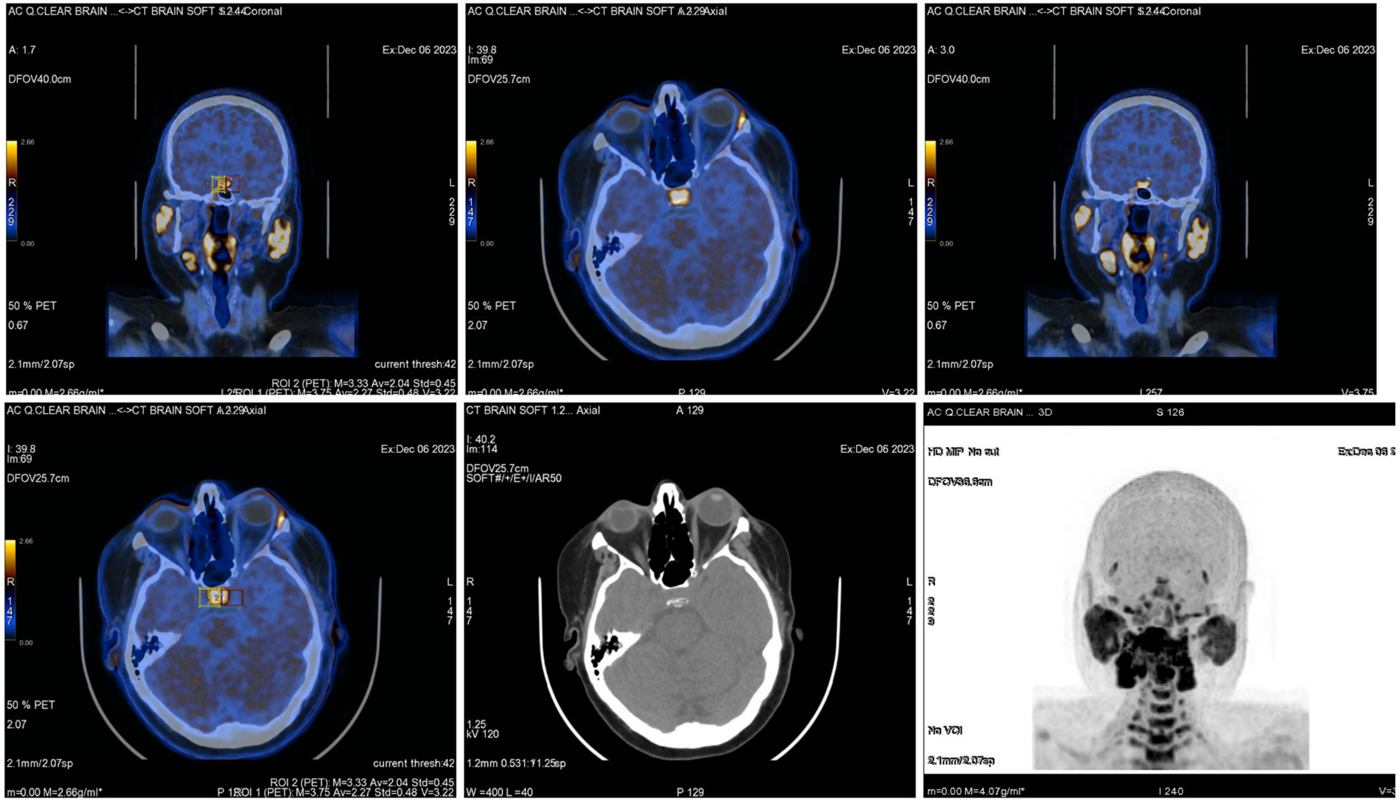

Suppl. Figure S3. [11C]C-MET PET/CT study - False positive. (Patient No. 12)

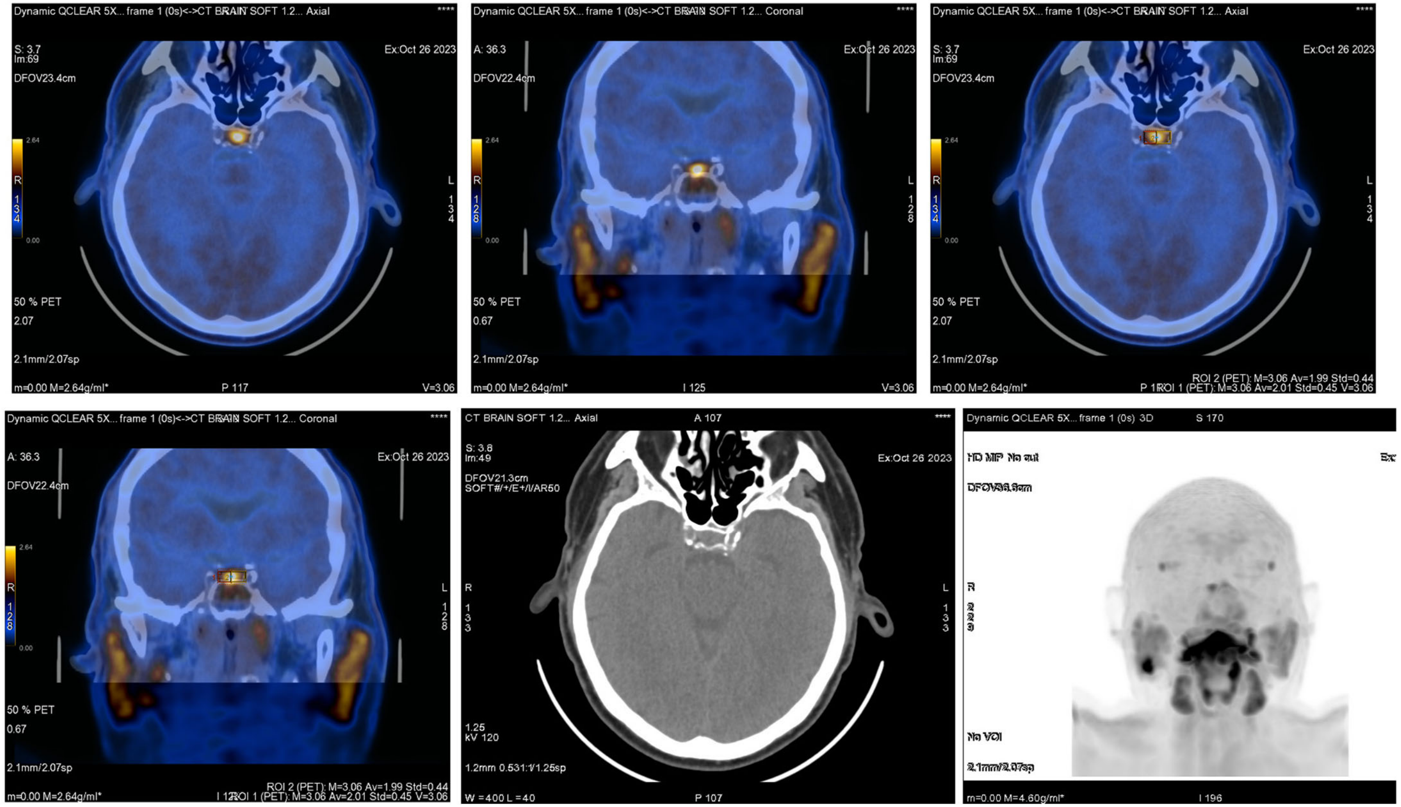

Suppl. Figure S4. [^{11}C]C-MET PET/CT study - False negative. (Patient No. 5)
